# Toward a Subject-Independent EEG-Based Neural Indicator of Task Proficiency During Training

**DOI:** 10.3389/fnrgo.2020.618632

**Published:** 2021-01-14

**Authors:** Bret Kenny, Sarah D. Power

**Affiliations:** ^1^Faculty of Medicine, Memorial University of Newfoundland, St. John's, NL, Canada; ^2^Faculty of Engineering and Applied Science, Memorial University of Newfoundland, St. John's, NL, Canada

**Keywords:** passive brain-computer interface, EEG, classification, spatial knowledge acquisition, cognitive state prediction, subject-independent

## Abstract

This study explores the feasibility of developing an EEG-based neural indicator of task proficiency based on subject-independent mental state classification. Such a neural indicator could be used in the development of a passive brain-computer interface to potentially enhance training effectiveness and efficiency. A spatial knowledge acquisition training protocol was used in this study. Fifteen participants acquired spatial knowledge in a novel virtual environment via 60 navigation trials (divided into ten blocks). Task performance (time required to complete trials), perceived task certainty, and EEG signal data were collected. For each participant, 1 s epochs of EEG data were classified as either from the “low proficiency, 0” or “high proficiency, 1” state using a support vector machine classifier trained on data from the remaining 14 participants. The average epoch classification per trial was used to calculate a neural indicator (NI) ranging from 0 (“low proficiency”) to 1 (“high proficiency”). Trends in the NI throughout the session—from the first to the last trial—were analyzed using a repeated measure mixed model linear regression. There were nine participants for whom the neural indicator was quite effective in tracking the progression from low to high proficiency. These participants demonstrated a significant (*p* < 0.001) increase in the neural indicator throughout the training from NI = 0.15 in block 1 to NI = 0.81 (on average) in block 10, with the average NI reaching a plateau after block 7. For the remaining participants, the NI did not effectively track the progression of task proficiency. The results support the potential of a subject-independent EEG-based neural indicator of task proficiency and encourage further research toward this objective.

## Introduction

A passive brain-computer interface (pBCI) is a system that enriches human-machine interaction by providing implicit information about a user's mental state (e.g., cognitive, affective) and adapting the environment accordingly (Brunner et al., 2015). Example applications include workload estimation in the workplace, feedback of mental states to improve wellness or manage stress, and enhanced product development (Baldwin and Penaranda, 2012). An example of the latter is “Intelligent Tutoring Systems (ITS)” (Chaouachi et al., 2015), which provide the user with an adapted and individualized learning environment. This adaptation can be operated with regards to several considerations (cognitive, emotional, etc.), and can be related to different aspects of the system's interaction strategy (e.g., selection of the next learning step, providing individualized feedback, etc.) (Zander and Kothe, 2011; Chaouachi et al., 2015). A pBCI for learning/training would support, for example, programs based on “mastery learning,” an effective method of instruction where a trainee must achieve proficiency in more fundamental, prerequisite knowledge prior to progressing to subsequent tasks of increasing difficulty and complexity (Block and Burns, 1976; Kulik et al., 1990). pBCIs would be useful for any software-based training programs, including immersive, experiential training based on simulation or virtual environments (Lécuyer et al., 2008).

The novel value of a pBCI for training programs would lie in its cognitive indicator of task proficiency. Traditionally, the only indicators that have been available to assess a trainee's task proficiency are their performance measures. However, according to the processing efficiency and attentional control theories, there is a crucial distinction between performance effectiveness (i.e., quality of performance) and processing efficiency [i.e., performance effectiveness divided by cognitive effort required to perform a task at that level Eysenck and Calvo, 1992]. Therefore, a trainee's performance may reach acceptable levels as indicated by performance measures alone before they are truly proficient in a skill; that is, before they are able to achieve good performance with little mental effort. Indeed, one aspect of expertise in a task is the development of automaticity (Ayaz et al., 2012), related to the idea that skill and mental workload are generally inversely related (Gopher and Kimchi, 1989). Particularly in high-risk or safety-critical occupations, it is crucial that personnel are able to perform their required tasks not just effectively but also as efficiently as possible, so that in a real-life scenario, where there will almost certainly be additional strains on cognitive resources vs. a simulated training condition, the individual will have developed automaticity and remain able to perform the task effectively. A truly effective training program would make use of an assessment measure that could capture this information about the trainee's cognitive state.

A key component of the processing efficiency and attentional control theories involves the relationship between anxiety, cognition, and task performance (Eysenck and Calvo, 1992; Derakshan and Eysenck, 2009). Anxiety in this context can be considered as an induced cognitive state when an individual's goal is threatened. Therefore, while individuals are training, the associated lack of confidence or certainty in their ability during the early stages of training can evoke a high-anxiety state which motivates individuals to improve their performance to avoid future negative task outcomes. Conversely, the confidence or certainty in task performance experienced by trainees late in a training protocol can satisfy an individual's desire to achieve a certain performance. Once an individual's desired task performance has been achieved, anxiety is reduced along with motivation to further improve task performance (Derakshan and Eysenck, 2009). The concept that anxiety adversely affects the attentional control of the working memory system is central to the attentional control theory. There is individual variance in the working memory system and thus individuals display differences in anxiety, cognition, and task performance. Broadly speaking, states of relative uncertainty are associated with the use of bottom-up processing (i.e., low processing efficiency), while states of relative certainty are associated with use of top-down processing (i.e., high processing efficiency) (Klimesch, 1999, 2012; Klimesch et al., 2006; Benedek et al., 2011). A cognitive measure capable of capturing this transition from an unskilled to a proficient state during a training protocol could be used to provide an optimized, personalized training experience for individual trainees.

Physiological metrics are often sensitive to aspects of a task that other metrics do not recognize (Baldwin and Penaranda, 2012), and thus have great potential to provide this important complementary information regarding a trainee's task proficiency. Indeed, current models of automaticity related to the development of expertise in certain tasks suggest that there are shifts in the functional neuroanatomy of task performance (Gopher and Kimchi, 1989). As automaticity develops in various tasks, attentional resources, largely associated with the prefrontal cortex, become available and can be devoted to perform other tasks (Ayaz et al., 2012). The development of expertise has been studied across a wide range of motor, visuomotor, perceptual, and cognitive tasks, and from diverse research perspectives (Ericsson, 2006). Four main patterns of practice-related activation change have been identified, including either (1) increased or (2) decreased activation in the brain areas involved in task performance, or (3) functional redistribution of brain activity, in which some initial areas of activation increase while others decrease, or (4) a functional reorganization of brain activity, in which the pattern of increasing and decreasing activation occurs across distinct brain areas in addition to the initial areas (Kelly and Garavan, 2005).

EEG is a non-invasive neuroimaging modality that measures transient electrical changes in the cortex associated with cognitive activity. EEG has been the focus of much BCI research, particularly for use in mental state prediction. A number of recent studies have investigated the use of EEG-based pBCIs to develop cognitive assessments of attention, workload, and fatigue for such tasks as car driving, air traffic control, aircraft piloting, helicopter piloting, laparoscopic surgery, and other cognitive tasks (Borghini et al., 2012a,b, 2015, 2016a,b, 2017a,b; Appel et al., 2019; Andreessen et al., 2020). A few EEG-based pBCI studies have explored neural indicators of training (Borghini et al., 2012b, 2016a,b, 2017a; Gerjets et al., 2014), but these have focussed on inter-session changes. Very little focus has been given to within-session indicators, which would be useful to support the “mastery learning” paradigm mentioned earlier. Furthermore, while multi-session training protocols certainly make the most sense for many tasks (e.g., aircraft piloting, air traffic control) since in practice these skills would be learned over long periods, some tasks—for example, emergency response procedures—must be learned much more quickly, often in a single session.

A recent study completed by the authors observed changes in cortical activity throughout a single-session spatial knowledge acquisition training protocol using EEG (Kenny et al., 2019). This task was selected because environmental familiarity is essential during safety training for high-risk environments (Stone et al., 2009) so that individuals will be able to act autonomously in the event of an emergency egress (Garling et al., 1983; Cliburn and Rilea, 2008). In this study, 15 participants acquired spatial knowledge via 60 navigation trials (divided into 10 blocks) in a novel virtual environment. The participant's time performance, perceived task certainty, and EEG signal data were collected for each trial. A machine learning approach in which the classification accuracies of EEG data from block 1 vs. blocks 2–10 (individually) were calculated, and used as a measure of how the data changed throughout the session—the higher the classification accuracy, the more dissimilar the data from a given block was from the initial block (i.e., when the individual's proficiency level was very low). We observed a significant upward trend in the classification accuracy, which stabilized after block 7 when the trials were arranged in the order they were completed, and after block 9 when arranged in increasing order of the participant's perceived task certainty (and secondarily, in order of completion). The results suggested that the participants' EEG signals continued to change after both the time performance and certainty ratings had plateaued, supporting the idea that neural signals could provide additional information about task proficiency that more conventional measures cannot.

These results were promising and provided insight into neural activity patterns during a single training session. To be able to use this within-subject, classification-based method as a neural indicator in a real passive BCI system while a user completes a training protocol, successive blocks of trials would have to be classified against the initial block of trials, until a plateau in accuracy is reached, which would indicate that the neural signals have stabilized and, presumably, that no further training-related changes are occurring. Given that blocks of multiple trials are needed to perform the classification, this method could be somewhat time-consuming, as the duration of blocks needed to adequately train the classifiers could be relatively long, and results from multiple blocks would be required to observe a plateau.

In this paper, we build on our previous work and explore a potential neural indicator of task proficiency that is much more time efficient. Specifically, we explore the possibility of classifying mental states related to task certainty associated with low and high task proficiency based on a subject-independent classifier trained on a database of “previous” trainees.

## Methodology

### Participants

Eighteen healthy volunteers were recruited to participate in this study. The data from three participants was not included in the analysis due to a software error, participant sickness during data collection, and excessive electromyographic (EMG) noise. Ultimately, fifteen participants were included in the analysis: nine male (27.9 ± 4.3 years, seven right-handed) and six female (26.2 ± 4.4 years, all right-handed). Participant inclusion criteria stipulated an age range of 18–65 years old, normal or corrected-to-normal vision, normal hearing, no history of neurological disease, disorder or injury, no cognitive impairment, no previous exposure to an offshore oil and gas platform (as this was the environment used in the experimental task), and significant experience using Xbox™ (or similar) controller. Participants were asked not to consume alcohol or caffeine for a period of 4 h before the experimental session. Participants of this study were recruited on a voluntary basis from the Memorial University campus in St. John's, NL, and self-selected based on the inclusion criteria. This study was approved by the Interdisciplinary Committee on Ethics in Human Research (ICEHR) board at Memorial University of Newfoundland, and all participants provided written informed consent prior to participating.

### Data Collection

The experimental protocol used in this study was previously reported in Kenny et al. (2019). Data from participants were collected on an individual basis during a single experiment session, ~2–3 h in duration. Three categories of data were collected during each experiment: neural, behavioral, and self-reported. The data were collected as participants performed 60 navigation trials within a virtual environment simulator in which they had to travel, unguided, from a starting location to a target location. Neural signals were collected throughout all trials via a 64-channel EEG system. Behavioral data were recorded as the time to complete each navigation trial. Self-reported data were recorded as the participant's perceived certainty of the target room location at the beginning of the trial.

### Virtual Environment—AVERT

Participants completed navigation trials using a desktop-based virtual environment simulator created by researchers at Memorial University. “AVERT: All-Hands Virtual Emergency Response Trainer” was developed for the purposes of improving safety training in the offshore oil industry (House et al., 2014). Specifically, AVERT is a life-like digital rendering of an offshore oil and gas platform that can be used to develop such skills as spatial knowledge, alarm recognition, mustering procedures, hazard avoidance, and emergency response (House et al., 2014; Musharraf et al., 2016). The virtual environment is displayed to participants via a desktop-user interface including a computer monitor and Xbox™ controller. The user has a first-person perspective in the virtual environment and can control head direction and translational movement via the XBox™ controller. The virtual environment is immersive and engaging; participants can interact with objects such as doors, and audio provided by two external speakers provides a realistic sense of space.

### Neural Data

A 64-channel EEG system (ActiChamp, BrainProducts, GmbH) was used to collect neural signals during the completion of navigation trials. Electrodes were arranged in the international 10–20 placement system and sampled at 500 Hz. The reference electrode and ground electrode were assigned as FCz and FPz, respectively. During the initial placement of the electrode cap on each participant, impedances were lowered below 15 kΩ. Subsequently, after every twelve trials, the electrode impedences were checked and re-set below 15 kΩ as necessary. Equipment setup procedures were accurately followed and participants received adequate education regarding data collection best practices (e.g., limiting movement).

### Behavioral Data

Participants' behavioral performance data were recorded as the time to complete each navigation trial. Two factors were controlled to reduce variance due to individual controller abilities and preferences. First, the walking/movement speed within AVERT was fixed to a constant value regardless of a participant's desired speed. Second, an elevator was used to travel between floors to simplify controls and to prevent simulation sickness caused by traveling down winding staircases. During pilot testing of this study, both of these factors were found to negatively affect performance regardless of navigation ability and spatial knowledge. Furthermore, participants were given time to practice using the controller to navigate through the virtual environment during a habituation phase, described below.

Time performance per trial was calculated as the percent difference from an “ideal” time performance. This normalized measure was used rather than just the raw time to complete the trial in order to account for different trial route lengths. The “ideal” time for each trial was set to be the time taken by one of the authors (who knew the locations of all rooms very well and could navigate between them immediately and without hesitation) less 15% to account for potential deficiencies of the author in controller usage.

### Perceived Task Certainty Data

Participants provided a self-reported task certainty rating by responding to the following question at the end of each trial: “At the beginning of the trial, did you know how to get to the target room location? Please rate on the scale (1–10) below how sure you were.” A rating of 1 indicated a low certainty of how to navigate to the target room, and a rating of 10 indicated a high certainty of how to navigate to the target room. The objective of this question was to gauge the level of perceived task certainty each participant had at the beginning of the trial, after being presented with the target room location. Therefore, this question does not assert how well one thought they *performed* in a given trial, but how certain they were at the beginning of the trial of the target room's location, regardless of how they ultimately performed. Participants were specifically asked to reflect on their level of certainty at the beginning of the trial as this best represents their cognitive state during the trial regardless of their behavioral performance. For example, participants could indicate an uncertain state at the beginning of the trial but correctly locate the target room, or conversely, they could indicate a certain state but ultimately be wrong about the target room location.

### Experiment Protocol

The experiment session was divided into three phases: habituation, introduction, and navigation. The results presented in this paper are based on data collected in the navigation phase of the session only.

#### Phase 1: Habituation

The first phase of the experiment was intended to expose participants to the virtual environment interface and to confirm controller skill level. Phase 1 was designed to reduce controller motor learning within the experiment. Participants were recruited who had significant previous experience using an Xbox™ controller. Additionally, controls to move through the environment were simple and included two joysticks, one each to control head and translational movement, and one button to interact with objects in the environment. Participants were introduced to AVERT and demonstrated their controller skill in a part of the virtual environment which was different than the environment used in phases 2 and 3. That is, participants remained naïve to the environment used in the spatial knowledge acquisition training protocol.

#### Phase 2: Introduction

The second phase of the experiment introduced participants to twelve rooms within the accommodations area of the virtual offshore oil and gas platform. Phase 2 was designed to provide participants with a single, brief exposure to each of these rooms via guided navigation. Pilot testing indicated that without this introduction, participants often spend an excessive amount of time performing trial-and-error searching for the target room. Additionally, such an introduction simulates a workplace “walk-through” that commonly takes place during basic safety training. Participants were told that the intention was not for them to remember the locations of all rooms within this phase, and that they would be given the opportunity to learn to navigate between the rooms in the following phase. The twelve rooms were evenly distributed across six floors of the accommodations area and were representative of common rooms found on an oil and gas platform (Table 1).

**Table 1:** The names and floor number of the twelve rooms used to complete navigation trials in phase 3 of the experiment protocol.

**Floor**	**Room name**	
1	Laundry room	Computer room
2	Quiet room	Kitchen
3	Maintenance	Fire room
4	Cabin	IT room
5	Electrical room	Office
6	Control room	Medic office

#### Phase 3: Navigation

The third phase of the experiment involved the collection of data as participants acquired spatial knowledge in an unfamiliar virtual environment. Participants completed sixty navigation trials, which required navigation between a start location and a target room. Note that all trials contained unique pairs of the twelve rooms presented in phase 2, and that participants were exposed to each room twice per twelve consecutive trials (once as the starting room, and once as the target room). Participants had a maximum of 3 min to perform each trial in order to limit over-exposure to the environment and wayfinding behavior. Phase 3 was designed such that through repeated exposures to the environment, participants could acquire sufficient spatial knowledge that by the end of the 60 trials, they could easily and accurately navigate between any two room locations. Several important design considerations were made in the third phase to select the number of rooms, routes, and trials each participant would perform. These design considerations are explained in detail in Kenny et al. (2019).

Neural, behavioral, and task certainty data were recorded during phase 3. EEG data were recorded during both navigation and baseline trials. Baseline trials were 1-min periods of rest during which the participant would sit quietly with their eyes either open or closed. The eyes-open trials were performed prior to each block of six navigation trials (for a total of ten eyes-open trials), while eyes-closed trials were performed at the beginning and end of the 60 trials (for a total of two eyes-closed trials). Each navigation trial began with the participant appearing in one of the twelve rooms, and the name of the target room was presented on the screen after 3 s. EEG recording started with the presentation of the target room location and ended when the participant reached the target room location or reached the maximum trial time limit (3 min). The time to complete each navigation trial, and the self-reported certainty data, were recorded at the end of each navigation trial.

### Data Analysis

#### Pre-processing

EEG signals of all participants were pre-processed identically, and all signal processing operations were performed on a participant's cumulative set of data. The following pre-processing steps were performed using the EEGLab Matlab toolbox (Delorme and Makeig, 2004):

1. Manual removal of electromyographic (EMG) noise
2. Down sampling from 500 to 256 Hz
3. Bandpass filtering from 1 to 50 Hz
4. Independent component analysis (ICA) to remove electrooculographic (EOG) artifacts.

#### Signal Power

The pre-processed EEG data were subsequently used to calculate a set of signal power features to be used for the classification analysis. First, pre-processed data were used to calculate a baseline-normalized power time-series in four frequency bands: theta, alpha-low1, alpha-low2, and alpha-high. The theta and alpha frequency bands were selected as there is evidence suggesting they reflect cognitive and memory performance (Klimesch, 1999). In addition, previous related works have found the theta and alpha frequency bands to be useful for neural indicators of performance (Borghini et al., 2012a,b, 2015, 2016a,b, 2017a,b). The frequency bands were established on an individual basis using each participants' individual alpha frequency (IAF) (Klimesch, 1999) defined as: theta band: (IAF −6 Hz) to (IAF −4 Hz), alpha-low1 band: (IAF −4 Hz) to (IAF −2 Hz), alpha-low2 band: (IAF −2 Hz) to (IAF), and alpha-high band: (IAF) to (IAF +2 Hz). Each participant's IAF was calculated as the average of the frequencies that had maximum power, between 8 and 15 Hz, in the two eyes-closed baseline trials from the beginning and end of the sixty navigation trials (the average of these two trials was used to help account for any drift in the IAF that may have occurred throughout the session). The filter-Hilbert method was used to obtain a power time-series for each frequency band, and the data were z-score normalized using the first eyes-open baseline trial of the session.

**Figure 1:**
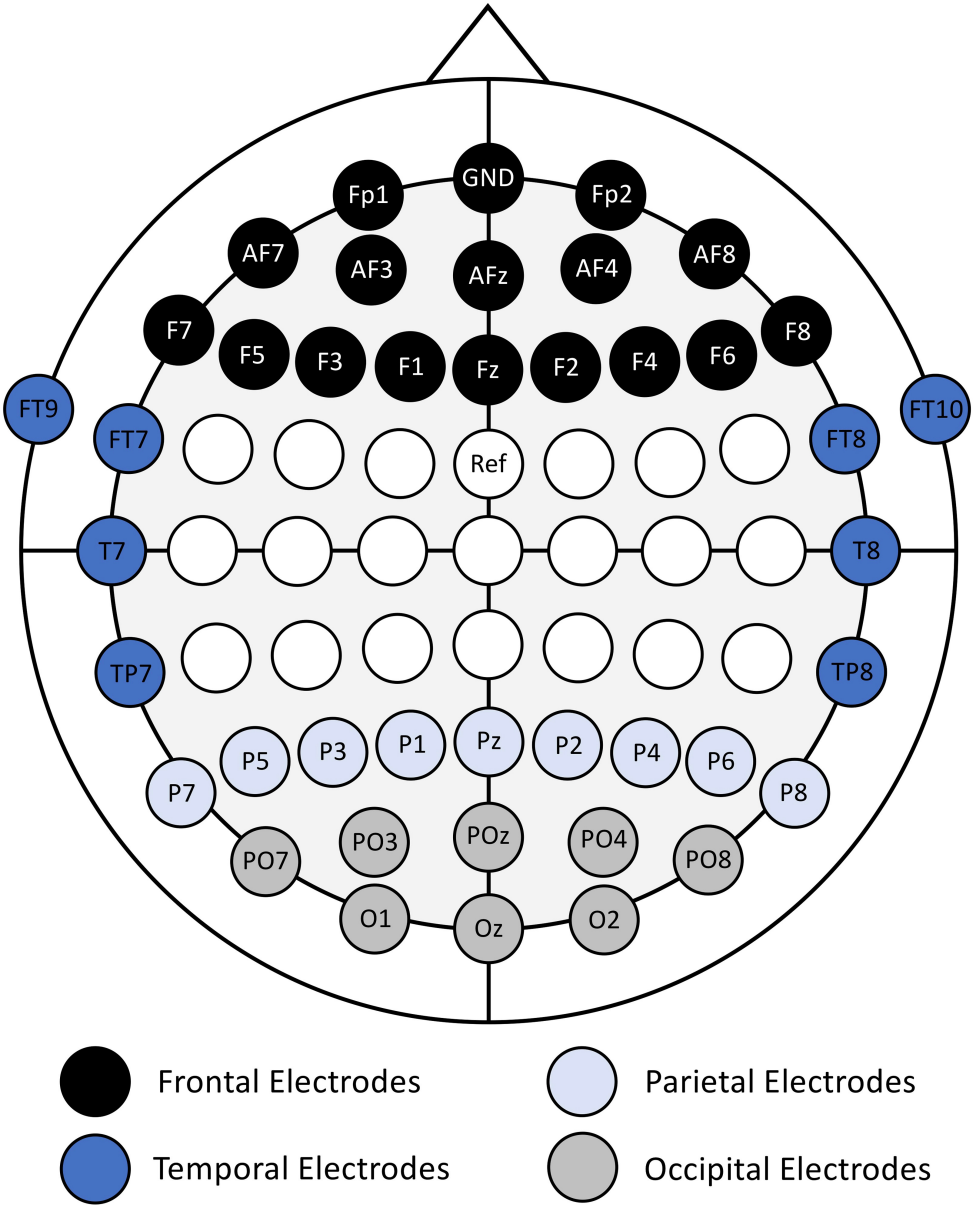
Electrode placement map with electrode names and cortical region labels (frontal, temporal, parietal, and occipital). Adapted from (Kenny et al., 2019).

The features used in the classification analyses were calculated using the normalized power time-series data from a subset of 41 EEG channels over the frontal, temporal, parietal, and occipital regions (Figure 1). The electrodes around the central sulcus were excluded to maintain consistency with our previous study (Kenny et al., 2019). The features were calculated as the average power in non-overlapping, 1 s windows (epochs), in each EEG channel and frequency band.

#### Classification

Based on the processing efficiency and attentional control theories discussed previously, we aimed to capture a neural indicator that would provide information about the trainee's processing efficiency. In practice, such a measure could then be used along with the trainee's performance effectiveness (as reflected by their behavioral performance measures) to get a more complete picture of their task proficiency. Based on our previous discussion of the processing efficiency and attentional control theories, we felt that the neural indicator should reflect how certain or confident a participant felt in their task execution. Our approach was therefore based on the classification of mental states associated with task certainty in the low and high proficiency states.

Training data were divided into classes reflecting “low task proficiency” and “high task proficiency” on a per participant basis by first arranging trials in order of increasing self-reported task certainty (secondarily, in order of completion). The bottom and top 10% of trials (i.e., 6 trials) were selected per participant to represent states of low and high task proficiency, respectively. Note that this approach to ordering the trials was taken rather than ordering simply by trial number to account for the possibility that an individual could by chance be (or feel) very proficient in a particular trial early in the session (e.g., if they happened to remember a room location from the habituation phase) or struggle with a trial late in the session. For each of the 15 participants, a classifier was trained on the data from the other 14 participants and tested using that participant's data. The minimum-redundancy maximum-relevance (mRMR) feature selection method (Peng et al., 2005), which is commonly used in EEG-based BCI studies (Lotte et al., 2018), was used to choose an optimal 10-dimensional feature set to perform classification using a support vector machine (SVM) classifier with a linear kernel. No data from the “test participant” were used in either feature selection or classifier training.

Note that because there were generally more samples (epochs) available in the “low proficiency” than the “high proficiency” state (due to participants taking longer to complete these trials), the number of samples per class that a given participant contributed to the training set were balanced via random sampling of the larger class (e.g., if there were 300 samples from the “low proficiency” class, but only 120 samples from the “high proficiency” class, then 120 samples from the “low proficiency” class were selected by random sampling for use in the classification analysis). However, the amount of data contributed by each participant to the training set was not necessarily equal. To minimize variance due to the random sampling of the larger class, 100 runs of the classification was performed for each participant, and the average classification accuracy was calculated over these 100 runs. Note that in each run the test data remained the same (i.e., all data from the “test participant”) while a new training set was selected via random sampling (to balance the classes within participants). The average number of samples per class per participant was 132 ± 28. The minimum was 80 samples/class.

#### Neural Indicator

A “neural indicator (NI)” (Equation 1) was calculated for each trial to indicate the degree to which a trial's epoched data was predicted as coming from a “low proficiency (0)” or “high proficiency (1)” cognitive state based on the classifier using the other participants' data. Specifically, for each trial, the average of the predicted class (i.e., 0 or 1) was calculated over all trial epochs. This would result in a value close to 0 if most epochs in a trial were classified as “low proficiency,” and a value close to 1 if most epochs in a trial were classified as “high proficiency.” We hoped to observe an increasing trend in this indicator from close to 0 in the early trials when the participant is not proficient in the task to close to 1 in later trials, as the participant became proficient in the task. The neural indicator, NI, was calculated for each trial, *t* according to:


(1) $$NI_{t}=\frac{\sum_{i=1}^{i=n_{t}}{\bar{c}}_{i}}{n_{t}}$$

where ${\bar{c}}_{i}$ is the predicted class of the *i*^*th*^ epoch of the trial, and *n*_*t*_ is the number of epochs in the trial.

### Statistical Analysis

To determine if the neural indicator changed significantly over the course of the training session as expected, a repeated measure mixed model linear regression with one within-subject factor (i.e., block #) was performed.

Furthermore, to determine if the neural indicator correlates with (1) the perceived certainty rating, and (2) the time performance, Pearson correlation coefficients were calculated for each participant. This was done over the entire session, as well as on data from just the beginning and end of the session (first and last 20% of data) where we expect the neural indicator to be most reliable.

## Results

### Perceived Certainty and Time Performance Data

**Figure 2:**
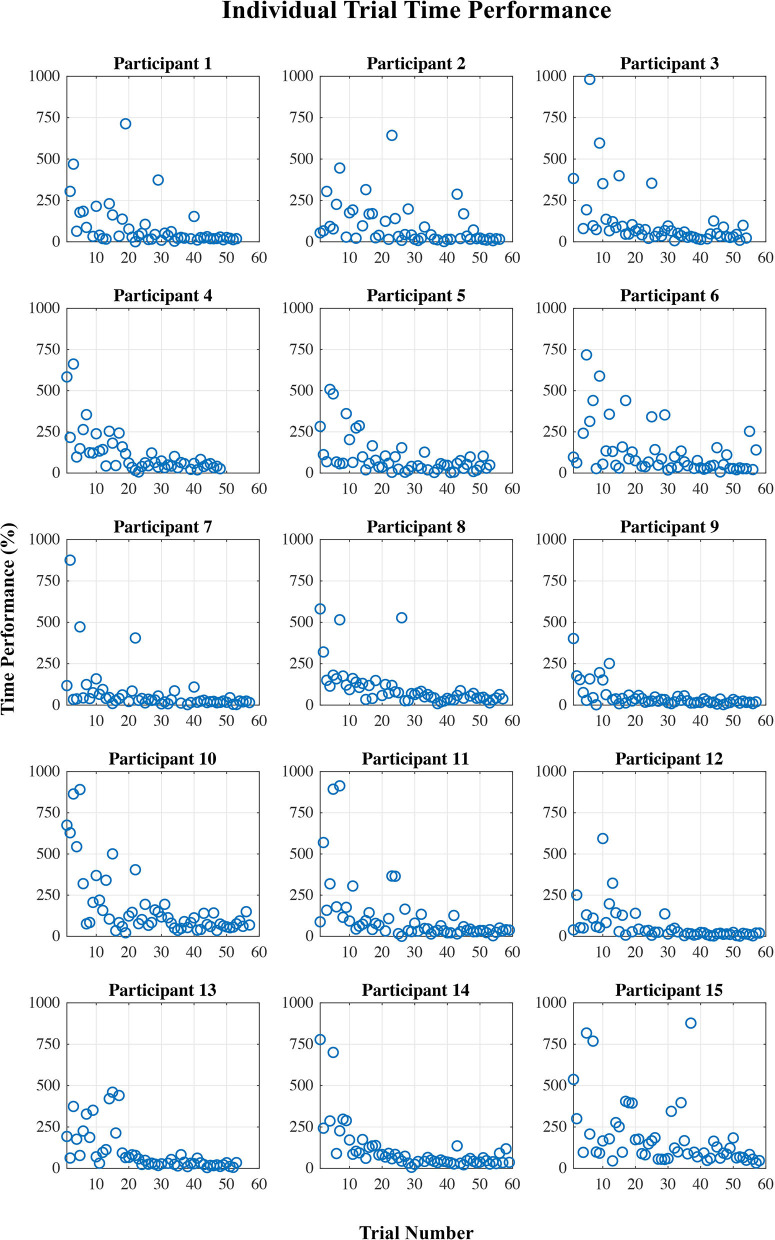
Time performance (%) per trial for each participant, calculated as the percent difference from an “ideal” time performance.

**Figure 3:**
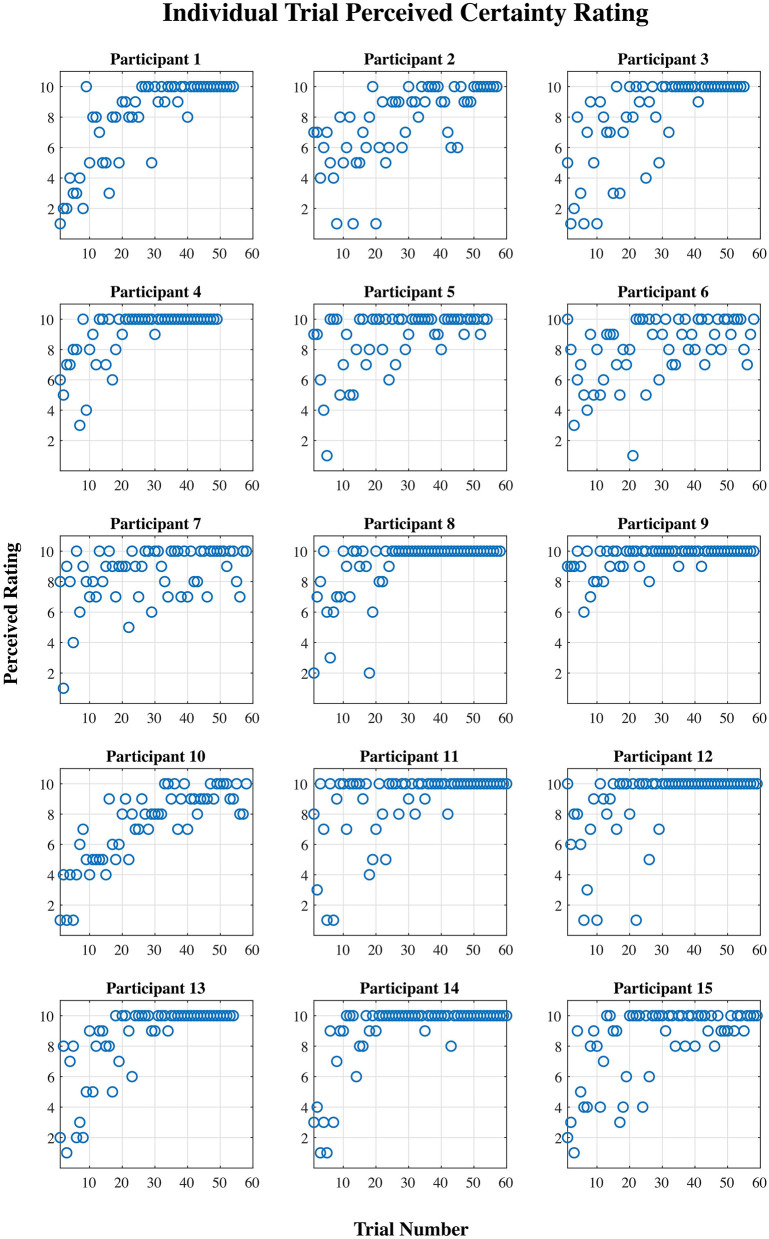
Perceived certainty rating (1–10) per trial for each participant.

Figures 2, 3 show plots of time performance vs. trial and perceived certainty rating vs. trial for all participants.

### EEG Classification-Based Neural Indicator (NI)

**Figure 4:**
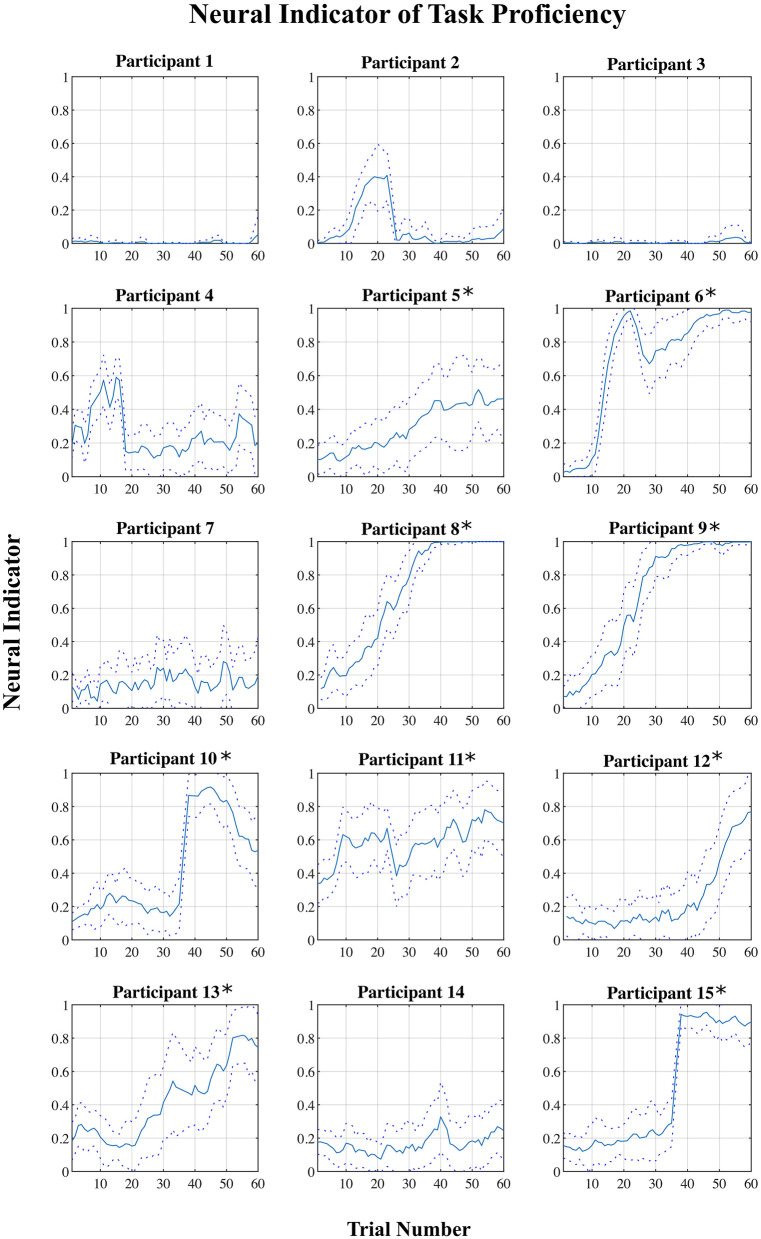
Mean neural indicator (NI) per trial for each participant. Dotted lines represent the 95% confidence interval. NI = 0 (“low proficiency”), NI = 1 (“high proficiency”). *Participants for which the NI was effective.

**Figure 5:**
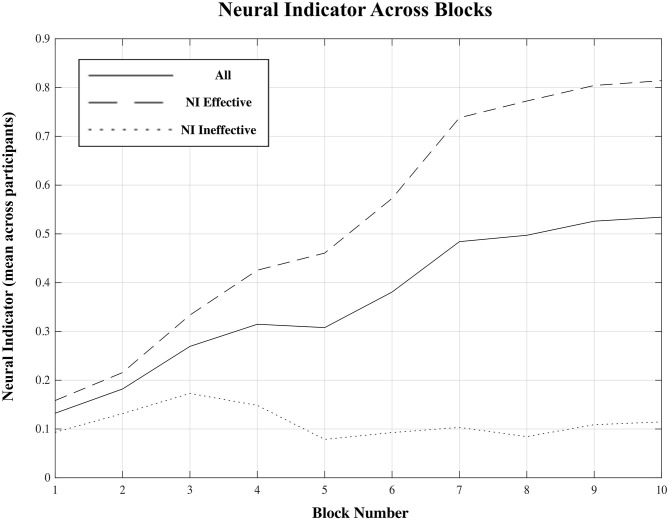
Mean neural indicator (NI) per block distinguishing the two subgroups for which the NI was effective and ineffective, as well as for all participants combined. NI = 0 (“low proficiency”), NI = 1 (“high proficiency”).

For each participant, a plot of the “neural indicator (NI)” (derived from the classification of each EEG signal epoch as representing a “low proficiency” or “high proficiency” state) across the 60 trials of the training session is shown in Figure 4. A plot of NI averaged across all participants, and across blocks of six consecutive trials, is shown in Figure 5.

Across all fifteen participants, the proposed “neural indicator” increased from NI = 0.13 in the first block to NI = 0.53 in the final block, as participants progressed from a “low proficiency” to a “high proficiency” state. A repeated measure mixed model linear regression analysis indicated a significant effect of block on neural indicator (β_Block_ = 0.029, t_(134)_ = 4.12, *p* < 0.001) across all participants.

However, based on the plots shown in Figure 4, there appears to be a division into two rather distinct groups—for nine out of fifteen participants (participants 5–6, 8–13, 15), the proposed measure follows the expected trend from “low proficiency” to “high proficiency” across the session blocks quite well, while for the remaining six participants (participants 1–4, 7, 14), no such trend exists and the NI remains relatively flat across the whole session. Plots of the average NI for each of these two distinct subgroups are also shown in Figure 5. For the group of participants for whom the measure appeared effective, the average NI increases significantly (repeated measures mixed model linear regression: β_Block_ = 0.065, t_(80)_ = 19.12, *p* < 0.001) from NI = 0.15 in the first block to NI = 0.81 in the final block. This means that on average 85% of epochs were classified as “low proficiency” in the first block, and 81% as “high proficiency” in the final block, for an overall accuracy of 83% in these two blocks. For the other subgroup, there was no significant increase in NI across the session (repeated measures mixed model linear regression: β_Block_ = 0.006, t_(53)_ = 1.48, *p* = 0.14), and the consistently low value of the NI indicates that a majority of epochs from a majority of trials across the session were classified as “low proficiency” for these participants. To assess the significance of our results, a non-parametric permutation test was applied to the overall classification accuracy of EEG epochs from Blocks 1 and 10 for each participant (Hajra et al., 2018). Specifically, we randomly redistributed the class labels for all EEG epochs for each participant, and then performed an identical classification procedure as we did with the correctly labeled data. This process was repeated 1,000 times, and the resulting accuracies were used to create a null distribution for each participant against which their true classification accuracy was compared. *P-*values were calculated as the proportion of accuracies from the null distribution that were greater than or equal to the true classification accuracy obtained. For each of the 9 participants from the “NI effective” group, classification accuracies were significantly greater than chance (*p* < 0.001) while for the other 6 participants they were not (*p* > 0.12).

### Feature Selection

**Figure 6:**
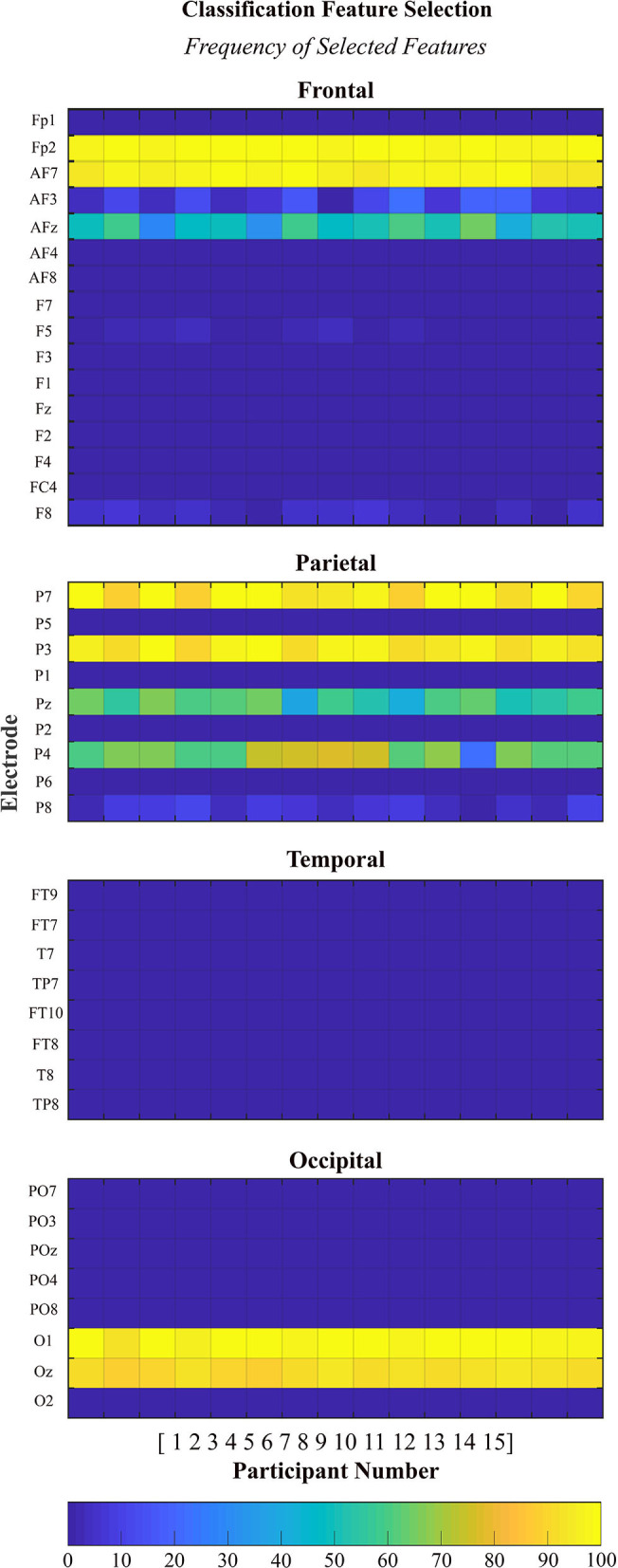
Histogram of features selected during classification. Each plot displays the absolute frequency of feature selection for features in the alpha-high frequency band organized by brain region (frontal, parietal, temporal, and occipital).

EEG signal features were selected and recorded for each run of the classification analyses for each participant. In each case, a total of 1,000 features were selected from the training data (10 dimentional feature set per run x 100 runs). The size of the feature pool was equal to the number of electrodes under consideration times the number of frequency bands (41 electrodes × 4 frequency bands = 164 features). The plots are organized by brain region (frontal, parietal, temporal, and occipital) and each plot contains the data from all 15 × 100 runs. The selected features were almost exclusively from the alpha-high frequency band and therefore only these results are presented, in Figure 6.

## Discussion

This study explored a potential neural indicator of task proficiency based on subject-independent EEG signal classification. The motivation of this work is to move toward the development of a neural indicator that could be used as a complementary assessment measure to enhance computer- or simulation-based training programs. The approach could be applied to a passive BCI in which a subject-independent classifier is trained on data from a group of “previous trainees” to track the transition from states of “low proficiency” to “high proficiency” of a “new trainee” whose data does not appear in the training set.

The results showed a division of the participants into two rather distinct groups—for nine out of fifteen participants, the proposed measure follows the expected trend from “low proficiency” to “high proficiency” across the session trials quite well, while for the remaining six participants, no such trend exists. This is supported by both the linear regression and correlation analyses (Table 2). There are some potential causes of the disparity in the results between the two groups. First, though, it is worth pointing out that variation in task performance and perceived task certainty among individual participants can be ruled out as a potential cause. All participants were naïve to the virtual environment prior to completing the training protocol, and all progressed through the session such that by the final block of trials, time performance and self-reported task certainty ratings were equivalent to that of a trained individual navigating the environment (see Figures 2, 3). Furthermore, our previously published results suggest that on average the cognitive activity of participants plateaued before the final block of trials (Kenny et al., 2019).

**Table 2:** Pearson correlation coefficient (r) between the neural indicator (NI) and (a) perceived certainty rating, and (b) time performance using the data from all trials, and the first and last 20% of trials.

**Participant**	**(a) Perceived certainty rating**		**(b) Time performance**	
	**All trials**	**First & Last 20% of trials**	**All trials**	**First & Last 20% of trials**
1	0.23 (*p* = 0.08)	−0.02 (*p* = 0.94)	0.05 (*p* = 0.70)	−0.04 (*p* = 0.87)
2	−0.19 (*p* = 0.14)	−0.02 (*p* = 0.94)	0.30 (*p* = 0.02)	0.04 (*p* = 0.85)
3	0.21 (*p* = 0.11)	0.27 (*p* = 0.25)	−0.06 (*p* = 0.67)	−0.20 (*p* = 0.40)
4	0.40 (*p* = 0.001)	−0.17 (*p* = 0.50)	0.35 (*p* = 0.01)	0.28 (*p* = 0.26)
5^*^	0.63 (*p* < 0.001)	0.51 (*p* = 0.01)	−0.30 (*p* = 0.02)	−0.54 (*p* = 0.01)
6^*^	0.52 (*p* < 0.001)	0.64 (*p* < 0.001)	−0.22 (*p* = 0.09)	−0.49 (*p* = 0.02)
7	0.32 (*p* = 0.01)	0.09 (*p* = 0.69)	−0.04 (*p* = 0.76)	0.04 (*p* = 0.86)
8^*^	0.69 (*p* < 0.001)	0.71 (*p* < 0.001)	−0.47 (*p* < 0.001)	−0.70 (*p* < 0.001)
9^*^	0.52 (*p* < 0.001)	0.62 (*p* = 0.001)	−0.51 (*p* < 0.001)	−0.64 (*p* = 0.001)
10^*^	0.61 (*p* < 0.001)	0.88 (*p* < 0.001)	−0.37 (*p* = 0.004)	−0.70 (*p* < 0.001)
11^*^	0.40 (*p* = 0.002)	0.59 (*p* = 0.003)	−0.47 (*p* < 0.001)	−0.60 (*p* = 0.002)
12^*^	0.32 (*p* = 0.01)	0.62 (*p* = 0.002)	−0.23 (*p* = 0.07)	−0.46 (*p* = 0.03)
13^*^	0.66 (*p* < 0.001)	0.75 (*p* < 0.001)	−0.35 (*p* = 0.01)	−0.66 (*p* = 0.001)
14	0.10 (*p* = 0.45)	0.30 (*p* = 0.16)	−0.06 (*p* = 0.64)	−0.21 (*p* = 0.32)
15^*^	0.51 (*p* < 0.001)	0.74 (*p* < 0.001)	−0.23 (*p* = 0.08)	−0.59 (*p* = 0.003)
Mean (all participants)	0.40 ± 0.24	0.43 ± 0.33	−0.17 ±0.26	−0.36 ± 0.32
Mean (“NI Effective”)	0.54 ± 0.12	0.67 ± 0.11	−0.35 ± 0.11	−0.60 ± 0.09
Mean (“NI Ineffective”)	0.18 ± 0.21	0.08 ± 0.18	0.09 ± 0.19	−0.01 ± 0.18

“NI effective” participants are indicated by*.

The present findings suggest that some of the participants demonstrated similar patterns of EEG activity as they transitioned through the training, allowing the classifier trained on the other participants' data to be effective for those individuals. On the other hand, for several other participants, it seems that their patterns of EEG activity must have been different from the majority and thus the classifier trained on the other participants was ineffective. Generalizability of a method based on a subject-independent classifier to all users could potentially be achieved if it incorporated a means of identifying and including only the subset of the training database that is sufficiently similar to that of the user to be classified. This may be difficult in this particular application, however, due to the fact that sample data from the user in the “high proficiency” state would not be available for comparison a priori. Future work should investigate whether data collected during training of a different task might be useful for this purpose.

Results from the authors' previous work (Kenny et al., 2019), which focused on subject-specific data analysis, provide some insight into the present results. In summary, the results of the authors' previous work noted several trends of average EEG signal power in the four frequency bands in consideration. Relevant to this study, on average, alpha-high power increased throughout the training session within multiple brain regions and was the dominant frequency band selected in feature selection and classification. Interestingly, the increase in alpha-high power was most significant with trials arranged in order of perceived task certainty rating as compared to in order of completion and in order of time performance. In that study, eleven participants demonstrated an increase in alpha-high power, while four participants demonstrated a decrease in alpha-high power, throughout the training (Kenny et al., 2019). A noteworthy finding was that of the six participants for whom the subject-independent classification method explored in the present study was not effective, three of them (participants 1, 2, and 4) demonstrated this decreasing alpha-high power trend, while of the nine participants for whom the method was effective, only one (participant 5) did.

The different individual alpha-high power trends as well as the individual subject-independent classification results are suggestive of individual differences in cognition and cognitive strategy exhibited to complete the training protocol. First, as previously discussed, according to the Processing Efficiency Theory, there is individual variation in working memory and task anxiety, which ultimately influences an individual's processing efficiency. For example, high anxiety individuals are more likely to increase their on-task effort (i.e., increase usage of cognitive processing resources) to avoid adverse outcomes (i.e., failure to locate the target room) as compared to low anxiety individuals (Eysenck and Calvo, 1992). The observed individual alpha-high power trends are also consistent with past studies that have explored the preferential use of different navigation strategies and thus, development of different representations of space (Hegarty et al., 2005). For example, egocentric and allocentric representations of space refer to procedural knowledge (turn-by-turn), and survey knowledge, respectively, which are often used when navigating an environment (Siegel and White, 1975; Montello, 1998; Ishikawa and Montello, 2006). Furthermore, egocentric and allocentric representations of space are also conceptually related to external (bottom-up) and internal (top-down) processing methods, as discussed above (Klimesch, 1999, 2012; Klimesch et al., 2006; Benedek et al., 2011). Individual differences in working memory and task anxiety and/or associated cognitive strategies are potential explanations for the different neural signals observed among participants throughout the training protocol.

A deeper look into the features selected via the automatic feature selection algorithm to perform the subject-independent classification analyses provides insight into some of the neural features that are *common* across many of the participants. Interestingly, the majority of features which were selected to be used for classification came from the alpha-high frequency band. Alpha-high power features were selected from the frontal (Fp2, AF7, and AFz), parietal (P7, P3, Pz, and P4), and occipital regions (O1 and Oz). No power features were selected from the temporal region in the alpha-high frequency band. This finding is consistent with the features selected to perform subject-specific classification as reported in Kenny et al. (2019). The selection of alpha-high features to perform the subject-independent classification supports the involvement of alpha oscillations in identifying the transition from bottom-up to top-down processing comparing trials of low and high proficiency states. Specifically, as reviewed by Klimesch (2012), there is great supporting evidence across multiple tasks that suggests increased alpha oscillations support the inhibition of other non-essential processes, including processing of task-irrelevant visual stimuli, which results in an increase in cognitive efficiency (i.e., top-down processing).

There have been a relatively small number of previous pBCI studies investigating neural indicators of task proficiency (Borghini et al., 2012a,b, 2015, 2016a,b, 2017a,b; Gerjets et al., 2014; Appel et al., 2019; Andreessen et al., 2020), and they have predominantly focused on complex tasks that are typically learned over long periods of time (e.g., aircraft flight tasks). Therefore, these studies have used methods to track proficiency across multiple sessions, making it impossible to compare these results to our study which is based on within-session analysis. For example, Borghini et al. (2017a) calculated a neural metric based on the stability of task-related brain activity across consecutive training sessions. Their results indicated that across the training sessions, cognitive stability and task performance improved and were highly correlated. To the best of our knowledge, the only other pBCI study investigating a neural indicator of task proficiency for use within a single training session was by our group (Biswas et al., 2020), in which the metric was based on a similar concept of cognitive stability. While results from this study were promising, with significant trends in the neural indicator observed over the course of the training session on average, issues with the appropriateness of the task used (specifically, that the task was too difficult for the majority of subjects to reach a sufficient level of task proficiency within a single session) made it difficult to confidently assess the effectiveness on an individual level. Note also that the other previous pBCI studies in this area have been based on subject-specific classification methods, and thus have the disadvantage of requiring calibration if used in a real scenario. The results of the present study add to the relatively limited body of literature regarding neural indicators of task proficiency by demonstrating that, at least for some participants, the transition from the low to high task proficiency states can be tracked quite effectively within a single, relatively short, training session using a subject-independent analysis.

A subject-independent neural indicator of task proficiency in training would have an important advantage over a subject-specific approach (either classification-based, or simply based on tracking alpha-high power, for example). Due to the inevitable correlation with time in the training application, in a subject-specific approach there is always the possibility that any observed trends are not training-related, but rather just due to other time-dependent changes in neural activity (e.g., frustration and fatigue). By using a classifier trained with data from a group of other individuals, such individual effects would be suppressed. The fact that we observed the expected trend in the proposed neural indicator based on a subject-independent classifier for a majority of the participants is encouraging. This could lead to a reliable cognitive indicator of task proficiency, by revealing when neural activity changes associated with training plateau. Since the neural indicator was based on classification of epochs into one of just two states, i.e., “low proficiency” and “high proficiency,” currently the value of this measure in the middle of the training session is likely unreliable to be used as an absolute measure of proficiency, and should only be used to indicate trends in the neural activity. This is supported by the correlation analysis, which show higher correlation values of the NI with both the perceived certainty rating at the extreme ends of the session as compared to across the whole session (for the “NI effective” group). Future work should involve increasing the reliability, accuracy, and precision of the measure throughout the session by incorporating a higher resolution prediction of proficiency. This would then facilitate further validation of the neural indicator. Also, further work must be done to improve the generalizability of the approach to all users.

### Study Limitations

The authors put forth considerable care and effort in the design and implementation of this study, however, there are limitations that should be noted. Firstly, while compelling results were obtained, the relatively small sample size limits the general applicability of the results to a larger population.

Secondly, the perceived task certainty rating was collected at the end of the trial, which could introduce participant response bias. Unfortunately, it was not possible to gather this data at the beginning of, or throughout, the trial without interrupting the participant's cognitive state. In addition, because there is just a single rating of certainty for each trial, all 1-s epochs within a given trial are given the same certainty label, though it is likely that the cognitive state of participants varied to some degree during each trial. Since the data was divided into two classes based on the perceived certainty rating, this means that the classification was based on somewhat uncertain labels. However, the trends in perceived certainty shown in Figure 3 make us quite confident in the division of data into low and high task proficiency samples for each individual.

Also, as mentioned above, the inter-subject variability observed in the results could have been influenced by potential differences in participant anxiety levels, which effects relevant cognitive processes. Though we indirectly measured each participant's task-related anxiety across trials through the “perceived certainty rating,” this information cannot be used to compare anxiety levels across participants. In future studies, having participants complete Form Y-2 of the State-Trait Anxiety Inventory (STAI) or similar, which assesses “trait anxiety” or anxiety-proneness, would be prudent to aid in interpretation of results.

The method of exposure to the spatial environment limits the generalizability of the results. Participants in this study acquired spatial knowledge via repeated route exposures in a novel virtual environment. These results could be different as compared to a method using other navigation methods, such as completely naïve wayfinding. Furthermore, it is not clear how these results would translate to different tasks.

Given the relatively small number of participants (*n* = 15), it is possible that over-fitting may have occurred, and in future the method should be verified on a larger sample.

Finally, the neuroimaging time window included EEG data collected during the completion of the single-session training protocol only. Previous studies have indicated that neural signals can continue to change over a period of hours/days following task practice.

## Conclusion

The long-term objective of this research is to develop a passive brain-computer interface to improve training outcomes through the incorporation of a cognitive metric of task proficiency to supplement existing behavioral and self-reported performance measures. Using a spatial knowledge acquisition task, we explored the feasibility of tracking a trainee's task proficiency throughout a training protocol using EEG data. The results of individual participants suggest that the use of a subject-independent classifier in this context is feasible, though further work is required to validate the method, and to increase reliability and generalizability. The application of this result could provide an improved training experience in computer-based and simulated training environments that is tailored to the individual needs of the trainee.

## Data Availability Statement

The datasets presented in this article are not readily available because release of study data will require approval of local ethics authority. Requests to access the datasets should be directed to Sarah D. Power, sd.power@mun.ca.

## Ethics Statement

This study was reviewed and approved by the Interdisciplinary Committee on Ethics in Human Research (ICEHR) Board—Memorial University of Newfoundland. The participants provided written informed consent to participate in this study.

## Author Contributions

BK: conceptualization, methodology, software, formal analysis, investigation, data curation, writing—original draft, and visualization. SP: conceptualization, methodology, formal analysis, resources, writing—review and editing, supervision, project administration, and funding acquisition. All authors contributed to the article and approved the submitted version.

## Conflict of Interest

Financial support for the conduct of the research was provided by the Research and Development Corporation of Newfoundland and Labrador (RDC) and the Natural Sciences and Engineering Research Council of Canada (NSERC). The sponsors did not have any involvement in the study design, data collection, analysis or interpretation, report writing, or the decision to submit the article for publication.
